# Esophagectomy or Total Gastrectomy for Siewert 2 Gastroesophageal Junction (GEJ) Adenocarcinoma? A Registry-Based Analysis

**DOI:** 10.1245/s10434-021-10346-x

**Published:** 2021-07-13

**Authors:** Sivesh K. Kamarajah, Alexander W. Phillips, Ewen A. Griffiths, Lorenzo Ferri, Wayne L. Hofstetter, Sheraz R. Markar

**Affiliations:** 1 Department of Upper Gastrointestinal Surgery, Queen Elizabeth Hospital Birmingham, University Hospitals Birmingham NHS Trust, Birmingham, UK; 2 Institute of Cancer and Genomic Sciences, College of Medical and Dental Sciences, University of Birmingham, Birmingham, UK; 3 Northern Oesophagogastric Unit, Royal Victoria Infirmary, Newcastle University Trust Hospitals, Newcastle-upon-Tyne, UK; 4 School of Medical Education, Newcastle University, Newcastle-upon-Tyne, Tyne and Wear, UK; 5 Division of Thoracic and Upper Gastrointestinal Surgery, Department of Surgery, McGill University Health Centre, Montréal, Quebec, Canada; 6 Department of Thoracic and Cardiovascular Surgery, University of Texas MD Anderson Cancer Center, Houston, TX USA; 7 Department of Surgery & Cancer, Imperial College London, London, UK; 8 Department of Molecular Medicine & Surgery, Karolinska Institutet, Stockholm, Sweden

## Abstract

**Backgrounds:**

Due to a lack of randomized and large studies, the optimal surgical approach for Siewert 2 gastroesophageal junctional (GEJ) adenocarcinoma remains unknown. This population-based cohort study aimed to compare survival between esophagectomy and total gastrectomy for the treatment of Siewert 2 GEJ adenocarcinoma.

**Methods:**

Data from the National Cancer Database (NCDB) from 2010 to 2016 was used to identify patients with non-metastatic Siewert 2 GEJ adenocarcinoma who received either esophagectomy (*n* = 999) or total gastrectomy (*n* = 8595). Propensity score-matching (PSM) and multivariable analyses were used to account for treatment selection bias.

**Results:**

Comparison of the unmatched cohort’s baseline demographics showed that the patients who received esophagectomy were younger, had a lower burden of medical comorbidities, and had fewer clinical positive lymph nodes. The patients in the unmatched cohort who received gastrectomy had a significantly shorter overall survival than those who received esophagectomy (median, 47 vs. 68 months [*p* < 0.001]; 5-year survival, 45 % vs. 53 %). After matching, gastrectomy was associated with significantly reduced survival compared with esophagectomy (median, 51 vs. 68 months [*p* < 0.001]; 5-year survival, 47 % vs. 53 %), which remained in the adjusted analyses (hazard ratio [HR], 1.22; 95 % confidence interval [CI], 1.09–1.35; *p* < 0.001).

**Conclusions:**

In this large-scale population study with propensity-matching to adjust for confounders, esophagectomy was prognostically superior to gastrectomy for the treatment of Siewert 2 GEJ adenocarcinoma despite comparable lymph node harvest, length of stay, and 90-day mortality. Adequately powered randomized controlled trials with robust surgical quality assurance are the next step in evaluating the prognostic outcomes of these surgical strategies for GEJ cancer.

**Supplementary Information:**

The online version contains supplementary material available at 10.1245/s10434-021-10346-x.

During the past decade, the distribution of esophageal cancer has been changing, with gastroesophageal junctional (GEJ) cancers becoming more prevalent,^1^ and multimodality therapy remains the cornerstone in the management of esophagogastric cancers.^2^ Staging of GEJ cancers is challenging due to the accuracy of clinical staging methods, which provide an overall accuracy of approximately 70 %.^3^

It is unclear whether esophagectomy or total gastrectomy is the optimal surgical approach for junctional cancers. Transthoracic esophagectomy (TTE) provides the benefit of a more extensive lymphadenectomy,^4^–^6^ which improves staging and is likely to have an impact on survival. Furthermore, TTE is associated with lower rates of R1 resections in proximal margins than gastrectomy, although distal margins may be at risk.^7^,^8^ However, TTE is associated with an increased incidence of pulmonary complications.^9^,^10^ The ultimate goal of surgery is to achieve a radical resection (R0) with adequate lymph node dissection, accompanied with minimal mortality and morbidity as well as optimal postoperative quality of life (QoL) with maximal survival.^11^

High-quality evidence on choice of surgery for Siewert 2 GEJ cancers is lacking. First, no randomized controlled trials (RCTs) in this area have been performed, although the ongoing CARDIA RCT, a multinational, prospective, randomized, clinical trial comparing transthoracic esophagectomy with transhiatal extended gastrectomy for Siewert 2 GEJ adenocarcinoma,^12^ may help to provide good-quality evidence on this topic. Second, two recent systematic reviews reported no difference in 5-year survival between esophagectomy and gastrectomy for GEJ cancers.^13^,^14^ Third, previous studies have not explored lymph node involvement in patients with Siewert 2 GEJ cancers. Therefore, the choice between esophagectomy and gastrectomy for Siewert 2 GEJ cancers in the absence of mediastinal lymph nodes remains unclear. However, the ongoing TIGER study will provide further understanding in this debate by establishing the pattern of lymph node spread in esophageal cancer.^15^

Heterogeneity exists within published retrospective studies because these studies often include distal esophageal and gastric cardia cancer.^16^ Furthermore, some studies have excluded patients after neoadjuvant therapy, making results difficult to interpret for patients with true GEJ cancer in the current era of greater use of neoadjuvant therapy. Therefore, the choice of esophagectomy or gastrectomy for GEJ cancers remains the subject of much debate and in the absence of robust evidence is largely driven by individual surgeon belief or preference.^16^,^17^

This study aimed to add further evidence to this debate by performing a national population-based cohort study to evaluate long-term survival outcomes for patients undergoing esophagectomy or gastrectomy using the National Cancer Data Base (NCDB) for Siewert 2 GEJ.^18^,^19^ Propensity-matched analysis was used to address treatment selection bias.

## Methods

### Data Source

The National Cancer Database (NCDB) is a project jointly sponsored by the Commission on Cancer (CoC) of the American College of Surgeons and the American Cancer Society.^20^,^21^ The NCDB gathers information from approximately 1500 CoC-accredited hospitals and includes more than 70 % of all newly diagnosed malignancies in the United States. It contains specific details about patient demographics (age, sex, race, insurance status), facility type and location, tumor characteristics (size, grade, stage, histology), treatment course (type of surgery, receipt of chemotherapy, and radiation therapy), and outcomes (resection margins, lymph node status, length of stay, short- and long-term mortality).

### Study Population

#### Inclusion Criteria

The study enrolled any patients with a non-metastatic Siewert 2 GEJ adenocarcinoma (Table S1) clinically staged according to the International Classification of Disease for Oncology, third edition (ICD-O-3) who received esophagectomy or gastrectomy between 2010 and 2016 in the de-identified NCDB.

#### Exclusion Criteria

The exclusion criteria ruled out other histology subtypes (e.g., squamous cell carcinoma, mucinous tumors, neuroendocrine tumors, and other histologies), patients who underwent endoscopic resection, other concurrent cancer diagnoses, and patients with metastatic and non-junctional esophageal cancer.

#### Study Definition

The following patient-level characteristics provided by NCDB were analyzed: age (18–35, 36–50, 51–65, 66–80, ≥81 years), race (white, other), Charlson-Deyo comorbidity score,^22^ year of diagnosis, insurance status (Medicare, Medicaid, private insurance, no insurance), zip code-level education status (<7 %, 7–12.9 %, 13–20.9 %, ≥21 %), zip code-level median household income (<$48,000, $48,000–62,999, ≥$63,000), and urban versus rural area of residence. The zip code-level education status represents the proportion of adults in the patient's zip code who did not graduate from high school and is categorized as equally proportioned quartiles among all U.S. zip codes. Hospital-level characteristics were analyzed in terms of facility type (academic, community, other), facility location (Midwest, Northeast, South, West), and hospital distance (<12.5, 12.5–49.9, ≥50.0 miles). Finally, we analyzed the following clinicopathologic characteristics: clinical T (T0-1, T2, T3-4, Tx) and N (N0, N+, Nx) status, tumor grade/differentiation (well/moderate, poor/anaplastic, unknown), margin status (positive, negative, unknown), and lymphovascular invasion (absent, present, unknown).

#### Selection of Siewert 2 Cancers

The NCDB provides location of cancers based on proximal and distal distance of tumor edge from incisors. Therefore, cancers arising within definitions of Siewert 2 cancers were included in the current analysis.

### Statistical Analysis

Categorical variables were compared using the chi-square test. Non-normally distributed data were analyzed using the Mann-Whitney *U* test. Survival was estimated using Kaplan-Meier survival curves and compared using the log-rank test. Multivariable analyses used Cox proportional hazards models. The conditional probability of receiving different treatment options (esophagectomy vs gastrectomy), as indicated by the propensity score, was estimated using a multivariable logistic regression model including all the variables listed in Table S2. Next, balanced cohorts using nearest-neighbor propensity score-matching (PSM) without replacement (caliper width 0.1 standard deviation) were developed.^23^ Balance diagnostics were performed using standardized mean differences, with a value lower than 0.1 indicating good balance.^23^ The overall survival (OS) of the matched patients who received the aforementioned treatment options was evaluated. A *p* value of lower than 0.05 was considered to be statistically significant. Data analysis was performed using R Foundation Statistical software (R 3.2.2) with TableOne, ggplot2, Hmisc, Matchit, and survival packages (R Foundation for Statistical Computing, Vienna, Austria), as previously reported.^24^

## Results

### Baseline Demographics

In this cohort, 9594 patients had GEJ adenocarcinoma, 999 (10 %) of whom received esophagectomy. The baseline demographics of the unmatched and matched cohorts are presented in Table 1. The median age of the entire cohort was 65 years (range, 18–90 years). The patients receiving gastrectomy were older (i.e., ≥80.4 % vs 2 %; *p* < 0.001), had a CDCC score of 2 or higher (2 % vs 1 %; *p* = 0.039), had clinical N2/N3 disease (10 % vs. 7 %; *p* < 0.001), and were less likely to have received neoadjuvant therapy (60 % vs 68 %; *p* < 0.001) or minimally invasive surgery (25 % vs. 33 %; *p* < 0.001).

**Table 1:** Baseline characteristics of patients with gastroesophageal junction adenocarcinoma in unmatched and matched cohorts

		Unmatched cohort			Matched cohort		
		Esophagectomy (*n* = 999) *n* (%)	Gastrectomy (*n* = 8595) *n* (%)	*p* Value	Esophagectomy (*n* = 999) *n* (%)	Gastrectomy (*n* = 3868) *n* (%)	*p* Value
Facility type	Community	295 (29.5)	3104 (36.1)	<0.001	295 (29.5)	1175 (30.4)	0.6
	Integrated	118 (11.8)	1371 (16.0)		586 (58.7)	2205 (57.0)	
	Academic	586 (58.7)	4120 (47.9)		118 (11.8)	488 (12.6)	
Facility location	Northeast	246 (24.6)	1986 (23.1)	<0.001	246 (24.6)	954 (24.7)	1.0
	Midwest	325 (32.5)	2159 (25.1)		325 (32.5)	1229 (31.8)	
	South	265 (26.5)	3127 (36.4)		265 (26.5)	1044 (27.0)	
	West	163 (16.3)	1323 (15.4)		163 (16.3)	641 (16.6)	
Hospital distance (miles)	<12.5	423 (42.3)	4080 (47.5)	<0.001	423 (42.3)	1651 (42.7)	0.8
	12.5–49.9	327 (32.7)	2897 (33.7)		327 (32.7)	1292 (33.4)	
	≥50	249 (24.9)	1618 (18.8)		249 (24.9)	925 (23.9)	
Year of diagnosis	2010–2011	224 (22.4)	2518 (29.3)	<0.001	224 (22.4)	883 (22.8)	1.0
	2012–2013	313 (31.3)	2316 (26.9)		313 (31.3)	1193 (30.8)	
	2014–2015	143 (14.3)	1317 (15.3)		143 (14.3)	559 (14.5)	
	2016–2017	319 (31.9)	2444 (28.4)		319 (31.9)	1233 (31.9)	
Age at diagnosis (years)	18–35	4 (0.4)	91 (1.1)	<0.001	4 (0.4)	22 (0.6)	0.8
	36–50	82 (8.2)	778 (9.1)		82 (8.2)	341 (8.8)	
	51–65	494 (49.4)	3547 (41.3)		494 (49.4)	1849 (47.8)	
	66–80	397 (39.7)	3788 (44.1)		397 (39.7)	1564 (40.4)	
	80+	22 (2.2)	377 (4.4)		22 (2.2)	92 (2.4)	
Sex	Male	878 (87.9)	6964 (81.0)	<0.001	878 (87.9)	3402 (88.0)	1.0
	Female	121 (12.1)	1631 (19.0)		121 (12.1)	466 (12.0)	
Race	White	971 (97.2)	7847 (91.3)	<0.001	971 (97.2)	3735 (96.6)	0.4
	Other	28 (2.8)	748 (8.7)		28 (2.8)	133 (3.4)	
CDCC score	0	665 (66.6)	5877 (68.4)	0.039	665 (66.6)	2566 (66.3)	1.0
	1–2	320 (32.0)	2514 (29.2)		320 (32.0)	1243 (32.1)	
	2+	14 (1.4)	204 (2.4)		14 (1.4)	59 (1.5)	
Insurance status	Medicare	454 (46.5)	4141 (49.0)	0.001	454 (45.4)	1755 (45.4)	0.8
	Medicaid	50 (5.1)	470 (5.6)		50 (5.0)	217 (5.6)	
	Private	455 (46.6)	3517 (41.6)		455 (45.5)	1721 (44.5)	
	Uninsured	17 (1.7)	321 (3.8)		40 (4.0)	175 (4.5)	
Education level	>21 %	210 (21.0)	1925 (22.4)	0.6	210 (21.0)	809 (20.9)	1.0
	13–20.9 %	227 (22.7)	1949 (22.7)		227 (22.7)	892 (23.1)	
	7–12.9 %	320 (32.0)	2780 (32.3)		320 (32.0)	1219 (31.5)	
	<7 %	242 (24.2)	1941 (22.6)		242 (24.2)	948 (24.5)	
Medical income	≤$47,999	356 (35.6)	2928 (34.1)	0.5	356 (35.6)	1337 (34.6)	0.8
	$48,000–62,999	254 (25.4)	2178 (25.3)		254 (25.4)	1001 (25.9)	
	$63,000+	389 (38.9)	3489 (40.6)		389 (38.9)	1530 (39.6)	
Residence	Metro	737 (73.8)	6777 (78.8)	0.001	737 (73.8)	2874 (74.3)	0.9
	Urban	182 (18.2)	1313 (15.3)		80 (8.0)	301 (7.8)	
	Rural	80 (8.0)	505 (5.9)		182 (18.2)	693 (17.9)	
AJCC clinical T stage	cT1	238 (23.8)	1618 (18.8)	<0.001	238 (23.8)	909 (23.5)	1.0
	cT2	236 (23.6)	1418 (16.5)		236 (23.6)	875 (22.6)	
	cT3	461 (46.1)	3634 (42.3)		461 (46.1)	1833 (47.4)	
	cT4	6 (0.6)	188 (2.2)		6 (0.6)	22 (0.6)	
	cTx	58 (5.8)	1737 (20.2)		58 (5.8)	229 (5.9)	
AJCC clinical N stage	cN0	553 (55.4)	4416 (51.4)	<0.001	553 (55.4)	2142 (55.4)	0.8
	cN1	349 (34.9)	2511 (29.2)		349 (34.9)	1314 (34.0)	
	cN2	59 (5.9)	705 (8.2)		59 (5.9)	254 (6.6)	
	cN3	11 (1.1)	144 (1.7)		11 (1.1)	37 (1.0)	
	cNx	27 (2.7)	819 (9.5)		27 (2.7)	121 (3.1)	
Neoadjuvant therapy	None	320 (32.0)	3423 (39.8)	<0.001	320 (32.0)	1269 (32.8)	0.7
	NCRT/NAC	679 (68.0)	5172 (60.2)		679 (68.0)	2599 (67.2)	
Surgical approach	Open	670 (67.1)	6491 (75.5)	<0.001	670 (67.1)	2699 (69.8)	0.1
	Minimally Invasive	329 (32.9)	2104 (24.5)		329 (32.9)	1169 (30.2)	

CDCC, Charlson-Deyo comorbidity; AJCC, American Joint Committee on Cancer; NCRT, neoadjuvant chemoradiotherapy; NAC, neoadjuvant chemotherapy

### Allocation to Esophagectomy

**Fig. 1:**
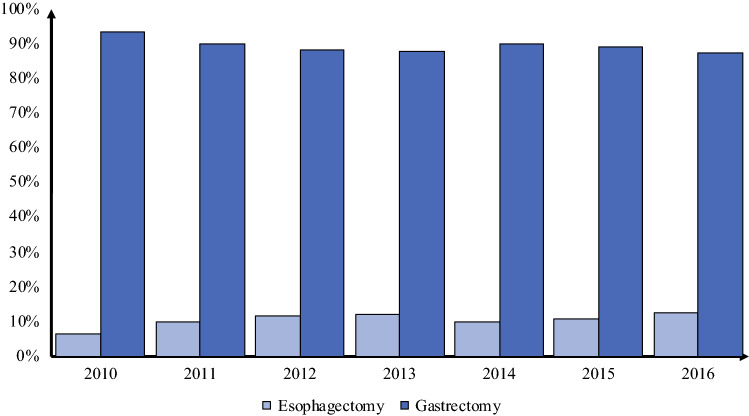
Trends in esophagectomy and gastrectomy for gastroesophageal junction adenocarcinoma.

In the unmatched cohort, multivariable logistic regressions analysis showed that the patients receiving gastrectomy were likely from community centers, to be male patients, to have a higher medical income, and to have advanced clinical T and N stage disease, and were less likely to have undergone neoadjuvant therapy or minimal access surgery (Table S3). During the study period, the rate of esophagectomy increased from 6 % in 2010 to 12 % in 2016 (Fig. 1). To account for this treatment selection bias, propensity score-matching was performed using the variables presented in Table S2. This resulted in well-balanced cohorts in terms of patient, tumor, and hospital demographics (Table 1). Standardized mean differences were calculated for each variable and ranged between 0.01 and 0.05, indicating a good balance.

### Pathologic and Postoperative Outcomes

In the matched cohorts, the patients who received esophagectomy had lower rates of pathologic T3/4 tumors (25 % vs. 38 %; *p* < 0.001) and N2/3 tumors (10 % vs. 18 %; *p* < 0.001) than the patients who received gastrectomy (Table 2). The patients who received esophagectomy had significantly higher margin-negative resections (94 % vs. 91 %; *p* = 0.001) and absence of lymphovascular invasion (57 % vs. 55 %; *p* < 0.001) than those who received gastrectomy. However, the rates of lymph node harvest were similar between the two groups (median, 14 vs. 15; *p* = 0.3). Also, no significant differences were observed in the length of stay (median, 9 vs. 9 days; *p* = 0.4), 30-day readmission (8 % vs. 8 %; *p* = 0.7), 30-day mortality (2 % vs. 2 %; *p* = 0.8), or 90-day mortality (5 % vs. 5 %; *p* = 0.4).

**Table 2:** Pathologic, oncologic, and surgical outcomes for patients with gastroesophageal junction adenocarcinoma in unmatched and matched cohorts

		Unmatched cohort			Matched cohort		
		Esophagectomy (*n* = 999) *n* (%)	Gastrectomy (*n* = 8595) *n* (%)	*p* Value	Esophagectomy (*n* = 999) *n* (%)	Gastrectomy (*n* = 3868) *n* (%)	*p* Value
Tumor grade	Well	69 (6.9)	626 (7.3)	0.8	69 (6.9)	300 (7.8)	0.8
	Moderate	402 (40.2)	3363 (39.1)		402 (40.2)	1534 (39.7)	
	Poor	412 (41.2)	3660 (42.6)		412 (41.2)	1588 (41.1)	
	Anaplastic	116 (11.6)	946 (11.0)		116 (11.6)	446 (11.5)	
AJCC pathologic T stage	pT0	164 (16.4)	818 (9.5)	<0.001	164 (16.4)	438 (11.3)	<0.001
	pT1	376 (37.6)	2195 (25.5)		376 (37.6)	1089 (28.2)	
	pT2	121 (12.1)	1321 (15.4)		121 (12.1)	605 (15.6)	
	pT3	249 (24.9)	3278 (38.1)		249 (24.9)	1398 (36.1)	
	pT4	1 (0.1)	333 (3.9)		1 (0.1)	86 (2.2)	
	pTx	88 (8.8)	650 (7.6)		88 (8.8)	252 (6.5)	
AJCC pathologic N stage	pN0	638 (63.9)	4518 (52.6)	<0.001	638 (63.9)	2202 (56.9)	<0.001
	pN1	179 (17.9)	1647 (19.2)		179 (17.9)	728 (18.8)	
	pN2	75 (7.5)	1110 (12.9)		75 (7.5)	463 (12.0)	
	pN3	27 (2.7)	693 (8.1)		27 (2.7)	235 (6.1)	
	pNx	80 (8.0)	627 (7.3)		80 (8.0)	240 (6.2)	
AJCC pathologic overall stage	Stage 0	230 (23.0)	1327 (15.4)	<0.001	230 (23.0)	624 (16.1)	<0.001
	Stage I	389 (38.9)	2631 (30.6)		389 (38.9)	1289 (33.3)	
	Stage II	99 (9.9)	1187 (13.8)		99 (9.9)	529 (13.7)	
	Stage III	281 (28.1)	3450 (40.1)		281 (28.1)	1426 (36.9)	
Regional nodes examined	Median (IQR)	14.0 (13.0)	14.0 (13.0)	0.6	14.0 (13.0)	15.0 (12.0)	0.3
Margin status	Positive	58 (5.8)	995 (11.6)	<0.001	58 (5.8)	358 (9.3)	0.001
	Negative	941 (94.2)	7600 (88.4)		941 (94.2)	3510 (90.7)	
Lymphovascular Invasion	Absent	573 (57.4)	4574 (53.2)	<0.001	573 (57.4)	2137 (55.2)	<0.001
	Present	146 (14.6)	2104 (24.5)		146 (14.6)	844 (21.8)	
	Unknown	280 (28.0)	1917 (22.3)		280 (28.0)	887 (22.9)	
Length of stay	Median (IQR)	9.0 (7.0)	9.0 (7.0)	0.7	9.0 (7.0)	9.0 (7.0)	0.4
30-Day mortality	No	977 (97.8)	8333 (97.0)	0.2	977 (97.8)	3774 (97.6)	0.8
	Yes	22 (2.2)	262 (3.0)		22 (2.2)	94 (2.4)	
90-Day mortality	No	953 (95.4)	8037 (93.5)	0.024	953 (95.4)	3663 (94.7)	0.4
	Yes	46 (4.6)	558 (6.5)		46 (4.6)	205 (5.3)	
30-Day readmission	No	917 (92.0)	7934 (92.5)	0.8	919 (92.0)	3565 (92.2)	0.7
	Yes - unplanned	14 (1.4)	107 (1.2)		14 (1.4)	42 (1.1)	
	Yes - planned	66 (6.6)	539 (6.3)		66 (6.6)	261 (6.7)	

AJCC, American Joint Committee on Cancer

### Survival Analyses

**Fig. 2:**
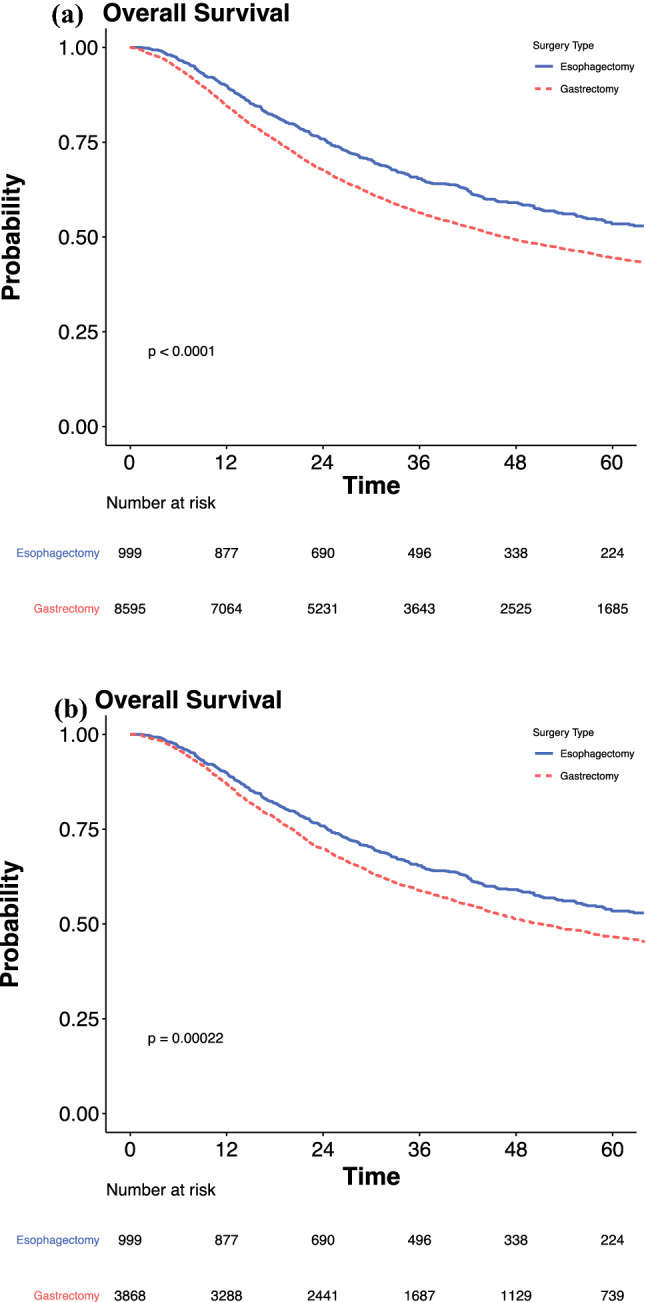
Overall survival after esophagectomy and gastrectomy for patients with Siewert 2 gastroesophageal junction adenocarcinoma in (**A**) unmatched and (**B**) matched cohorts.

In the unmatched cohort, the patients who received gastrectomy had significantly poor survival than those who received esophagectomy (median, 47 vs. 69 months; hazard ratio [HR], 1.19; 95 % confidence interval [CI], 1.07–1.31; *p* = 0.001; Fig. 2A; Table 3). In the matched cohort, gastrectomy resulted in a significantly shorter survival than esophagectomy (median, 51 vs. 68 months; HR, 1.22; 95 % CI, 1.09–1.35; *p* < 0.001; Fig. 2B; Table 1). The corresponding 5-year survival rate was 53 % for esophagectomy and 47 % for gastrectomy (*p* < 0.001). A sensitivity Cox regression analysis performed to account for pathologic tumor stage confirmed similar findings of a shorter survival with gastrectomy than with esophagectomy (HR, 1.13; 95 % CI, 1.01–1.26; *p* = 0.033; Table S4).

**Table 3:** Overall survival after esophagectomy and gastrectomy for patients with Siewert 2 gastroesophageal junction adenocarcinoma in unmatched and matched cohorts

Surgery type	Median survival Months (IQR)	HR (95 % CI)	*p* Value
Unmatched cohort			
Esophagectomy	68.1 (59.9–79.0)	Reference	0.001
Gastrectomy	46.6 (44.4–48.8)	1.19 (1.07–1.31)	

Matched cohort			
Esophagectomy	68.1 (59.9–79.0)	Reference	<0.001
Gastrectomy	51.1 (47.5–56.2)	1.22 (1.09–1.35)	

HR, hazard ratio; CI, confidence interval

## Discussion

This national population-based cohort study from the United States demonstrated that patients who received esophagectomy had a significantly longer survival than those who underwent gastrectomy for Siewert 2 GEJ adenocarcinoma. The rates of margin-negative resections were significantly higher with esophagectomy than with gastrectomy. However, postoperative morbidity and mortality and lymph node harvest results did not differ significantly between these two approaches. These findings provide clinical data contributing to clinical decision-making by suggesting that esophagectomy is superior to total gastrectomy for patients with GEJ cancer.

To date, the evidence supporting either esophagectomy or total gastrectomy for GEJ adenocarcinoma remains heterogeneous and limited. First, no RCTs comparing esophagectomy and gastrectomy for Siewert 2 GEJ adenocarcinoma exist. One RCT of patients with type 2 or 3 GEJ adenocarcinoma comparing left thoracoabdominal (*n* = 85) and transhiatal (*n* = 82) approaches found no significant difference in 5-year survival (38 % vs. 52 %), although the left thoracoabdominal approach had higher morbidity.^25^ However, this study may have been underpowered to detect a statistically significant difference between the two approaches. The ongoing CARDIA RCT may help to provide high-quality evidence for Siewert 2 tumors.

Second, two published systematic reviews^13^,^14^ reported comparable 5-year survival rates between esophagectomy (30–42 %) and gastrectomy (18–38 %) for GEJ, with acceptable rates for R0 resections and lymph node harvest. Early reports from Siewert et al. in the first decade of this century reported no significant difference between esophagectomy and extended total gastrectomy for Siewert 2 GEJ adenocarcinoma.^26^,^27^ A recent Dutch Upper Gastrointestinal Cancer Audit (DUCA) study demonstrated similar 3-year overall survival rates for esophagectomy and gastrectomy (36 % vs. 28 %). Notably, 90 % of the patients in the DUCA study underwent esophagectomy across different Siewert type GEJ cancers. Another study from Koeter et al.^16^ demonstrated that the choice of surgical approach (i.e., esophagectomy or gastrectomy) did not improve 5-year overall survival (36 % vs 33 %), but that administration of neoadjuvant therapy remained prognostic in adjusted outcomes.^16^ These previous studies, however, had a high-degree of heterogeneity due to (1) no reporting of neoadjuvant therapy administration, (2) inclusion of distal esophageal (type 1) or cardia/gastric (type 3) cancers and thus no focused analyses of type 2 GEJ adenocarcinomas, (3) selection bias between patients who received esophagectomy or gastrectomy, which may reflect either institutional policies (i.e., thoracic surgeons likely performing esophagectomy vs general or abdominal surgeons performing gastrectomy) or patient fitness (i.e., patients with borderline fitness who may have received an extended gastrectomy), and (4) variability in reporting of long-term survival, rendering the comparison with our results difficult.

One of the fundamental questions regarding GEJ tumors is the extent of lymphadenectomy required to achieve the best oncologic outcome.^5^,^6^ For patients whose staging suggests definite mediastinal nodes, understandably, an esophagectomy with two-field lymphadenectomy is likely to confer the greatest survival advantage.^28^ However, for patients whose lymph node metastases may not be apparent during clinical staging, further disease behavior and the spread of micrometastases, which may contribute to disease recurrence, are difficult to predict. It may be that a more extensive lymphadenectomy including mediastinal nodes provides an advantage even for type 3 tumors, which generally are regarded as gastric in origin. Therefore, this has been a matter of debate from previously published retrospective studies that may affect decision-making between esophagectomy or total gastrectomy.^29^–^33^ A recent prospective study from Japan investigated the incidence of lymph node metastases in each lymph node station in patients with a GEJ tumor. This study demonstrated a rate of lymph node metastases higher than 10 % in stations 1, 2, 3, 7, 9, and 11p, and in at least one of the lower mediastinal lymph node stations. Furthermore, subtotal esophagectomy with dissection of lymph nodes in the upper mediastinal station should be considered if esophageal involvement exceeds 4 cm, and lymph nodes lower mediastinal station should be dissected if esophageal involvement exceeds 2 cm.^34^ Although, the absolute lymph node harvest in the current study was similar between esophagectomy and gastrectomy, detailed information on lymph node metastasis location, extent of lymphadenectomy, and quality of radical resection is not reported in the NCDB, limiting a detailed analysis of these factors. The lymph nodes and tissue left inside the patient after lymphadenectomy are potentially more important that what is assessed in the pathologic specimen. This has been the Achilles heel of previous cancer resection studies assessing the prognostic impact of lymphadenectomy or surgical approach. Future studies, both observational cohort studies and randomized controlled trials, must seek to address this, with pictures or videos at the end of the lymphadenectomy providing an accurate measurement of intraoperative findings as well as a measure of quality of surgery and lymphadenectomy.^35^

This study had important limitations. First, a clear treatment selection bias existed between the patients receiving esophagectomy and those receiving gastrectomy, with surgeon preference often an unmeasured but crucial driving factor. This could have been due either to institutional policy (e.g., thoracic units favoring esophagectomy and abdominal units favoring gastrectomy) or to selection of patients with borderline fitness or cardiorespiratory disease for abdominal rather than thoracoabdominal resections. This study used propensity score-matching to adjust for several important variables, but additional relevant information that could affect treatment choice may have been missed. For example, patient comorbidities are recorded in the Charlson-Deyo score, but detailed data on specific comorbidities and overall functional status are not available in the dataset, a limitation shared by most national datasets. Second, and importantly, granular data regarding staging methods are not available in the NCDB. Hence, this study could not evaluate the proportion of patients staged with endoscopic ultrasound (EUS), laparoscopy, or positron emission tomography (PET) alone before esophagectomy or gastrectomy. As such, the study had a risk of misclassification between junctional tumors. Third, the NCDB does not distinguish whether longitudinal (proximal and distal) or circumferential margins were involved nor the type of gastrectomy (i.e., total or extended total), prohibiting ability to assess their relative importance.^36^ Fourth, classification of Siewert GEJ tumors in any national dataset is extremely challenging, and validation of this previously has been almost impossible. However, the current analysis attempted to justify methodology based on available data in the NCDB. Finally, this study was unable to capture data on long-term complications such as anastomotic strictures, recurrence (i.e., local or regional), and patient quality of life after esophagectomy and gastrectomy. For some patients, quality of life may be an important consideration when weighing their decision as to which treatment is best for them,^37^–^39^ warranting a stronger focus on this area in future studies investigating this topic.

In conclusion, a large-scale population study with propensity-matching to adjust for known confounders demonstrated that esophagectomy was prognostically superior to gastrectomy for the treatment of Siewert 2 GEJ adenocarcinoma despite comparable lymph node harvest, length of stay, and 90-day mortality. Adequately powered randomized controlled trials with robust surgical quality assurance are the next step to evaluate the prognostic outcomes of these surgical strategies for GEJ cancer.

## Supplementary Information

Below is the link to the electronic supplementary material.

Supplementary file1 (DOCX 34 kb)

## References

[1] Bartel M, Brahmbhatt B, Bhurwal A. Incidence of gastroesophageal junction cancer continues to rise: analysis of Surveillance, Epidemiology, and End Results (SEER) database.* J Clin Oncol.* 2019;37(4_suppl):40. 10.1200/JCO.2019.37.4_suppl.40.

[2] Kamarajah SK, Phillips AW, Hanna GB, Low DE, Markar SR. Definitive chemoradiotherapy compared to neoadjuvant chemoradiotherapy with esophagectomy for locoregional esophageal cancer: national population-based cohort study. *Ann Surg.* 2020. 10.1097/SLA.0000000000003941. 10.1097/SLA.0000000000003941 32865948

[3] Grotenhuis BA, Wijnhoven BP, Poley JW, Hermans JJ, Biermann K, Spaander MC (2013). Preoperative assessment of tumor location and station-specific lymph node status in patients with adenocarcinoma of the gastroesophageal junction. World J Surg..

[4] Kodama I, Kofuji K, Yano S, Shinozaki K, Murakami N, Hori H (1998). Lymph node metastasis and lymphadenectomy for carcinoma in the gastric cardia: clinical experience. Int Surg..

[5] Phillips AW, Lagarde SM, Navidi M, Disep B, Griffin SM (2017). Impact of extent of lymphadenectomy on survival, post neoadjuvant chemotherapy, and transthoracic esophagectomy. Ann Surg..

[6] Phillips AW, Hardy K, Navidi M, Kamarajah SK, Madhavan A, Immanuel A, Griffin SM (2019). Impact of extent of lymphadenectomy on survival, post neoadjuvant chemotherapy, and transthoracic esophagectomy. Ann Surg..

[7] Mariette C, Castel B, Toursel H, Fabre S, Balon JM, Triboulet JP (2002). Surgical management of and long-term survival after adenocarcinoma of the cardia. Br J Surg.

[8] Ito H, Clancy TE, Osteen RT, Swanson RS, Bueno R, Sugarbaker D (2004). Adenocarcinoma of the gastric cardia: what is the optimal surgical approach?. J Am Coll Surg..

[9] Hulscher JB, Tijssen JG, Obertop H, van Lanschot JJ (2001). Transthoracic versus transhiatal resection for carcinoma of the esophagus: a meta-analysis. Ann Thorac Surg..

[10] Straatman J, van der Wielen N, Cuesta MA, Daams F, Roig Garcia J, Bonavina L (2017). Minimally invasive versus open esophageal resection: three–year follow-up of the previously reported randomized controlled trial: the TIME trial. Ann Surg..

[11] Barbour AP, Rizk NP, Gonen M, Tang L, Bains MS, Rusch VW (2007). Adenocarcinoma of the gastroesophageal junction: influence of esophageal resection margin and operative approach on outcome. Ann Surg..

[12] Leers JM, Knepper L, van der Veen A, Schroder W, Fuchs H, Schiller P (2020). The CARDIA-trial protocol: a multinational, prospective, randomized, clinical trial comparing transthoracic esophagectomy with transhiatal extended gastrectomy in adenocarcinoma of the gastroesophageal junction (GEJ) type II. BMC Cancer..

[13] Heger P, Blank S, Goossen K, Nienhuser H, Diener MK, Ulrich A (2019). Thoracoabdominal versus transhiatal surgical approaches for adenocarcinoma of the esophagogastric junction: a systematic review and meta-analysis. Langenbecks Arch Surg..

[14] Haverkamp L, Ruurda JP, van Leeuwen MS, Siersema PD, van Hillegersberg R (2014). Systematic review of the surgical strategies of adenocarcinomas of the gastroesophageal junction. Surg Oncol..

[15] Hagens ERC, van Berge Henegouwen MI, van Sandick JW, Cuesta MA, van der Peet DL, Heisterkamp J (2019). Distribution of lymph node metastases in esophageal carcinoma [TIGER study]: study protocol of a multinational observational study. BMC Cancer..

[16] Koeter M, Parry K, Verhoeven RH, Luyer MD, Ruurda JP, van Hillegersberg R (2016). Perioperative treatment, not surgical approach, influences overall survival in patients with gastroesophageal junction tumors: a nationwide, population-based study in The Netherlands. Ann Surg Oncol..

[17] Haverkamp L, Seesing MF, Ruurda JP, Boone J, Hillegersberg RV (2017). Worldwide trends in surgical techniques in the treatment of esophageal and gastroesophageal junction cancer. Dis Esophagus..

[18] Reeh M, Mina S, Bockhorn M, Kutup A, Nentwich MF, Marx A (2012). Staging and outcome depending on surgical treatment in adenocarcinomas of the oesophagogastric junction. Br J Surg..

[19] Peters CJ, Hardwick RH, Vowler SL, Fitzgerald RC, Oesophageal Cancer Clinical and Molecular Stratification Study Group (2009). Generation and validation of a revised classification for oesophageal and junctional adenocarcinoma. Br J Surg..

[20] Bilimoria KY, Bentrem DJ, Ko CY, Ritchey J, Stewart AK, Winchester DP, Talamonti MS (2007). Validation of the 6th-edition AJCC pancreatic cancer staging system: report from the National Cancer Database. Cancer..

[21] Merkow RP, Rademaker AW, Bilimoria KY (2018). Practical guide to surgical data sets: National Cancer Database (NCDB). JAMA Surg..

[22] Quan H, Li B, Couris CM, Fushimi K, Graham P, Hider P (2011). Updating and validating the Charlson Comorbidity Index and score for risk adjustment in hospital discharge abstracts using data from 6 countries. Am J Epidemiol..

[23] Austin PC (2014). The use of propensity score methods with survival or time-to-event outcomes: reporting measures of effect similar to those used in randomized experiments. Stat Med..

[24] Kamarajah SK, Sonnenday CJ, Cho CS, Frankel TL, Bednar F, Lawrence TS (2021). Association of adjuvant radiotherapy with survival after margin-negative resection of pancreatic ductal adenocarcinoma: a propensity-matched National Cancer Database (NCDB) analysis. Ann Surg.

[25] Sasako M, Sano T, Yamamoto S, Sairenji M, Arai K, Kinoshita T (2006). Japan clinical oncology Group. Left thoracoabdominal approach versus abdominal-transhiatal approach for gastric cancer of the cardia or subcardia: a randomised controlled trial. Lancet Oncol.

[26] Stein HJ, Feith M, Siewert JR (2000). Cancer of the esophagogastric junction. Surg Oncol..

[27] Feith M, Stein HJ, Siewert JR (2006). Adenocarcinoma of the esophagogastric junction: surgical therapy based on 1602 consecutive resected patients. Surg Oncol Clin North Am..

[28] Mitchell KG, Ikoma N, Nelson DB, Maru DM, Erasmus JJ, Weston BR (2019). Mediastinal nodal involvement after neoadjuvant chemoradiation for Siewert II/III adenocarcinoma. Ann Thorac Surg..

[29] Yoshikawa T, Takeuchi H, Hasegawa S, Nozaki I, Kishi K, Ito S (2016). Theoretical therapeutic impact of lymph node dissection on adenocarcinoma and squamous cell carcinoma of the esophagogastric junction. Gastric Cancer..

[30] Kurokawa Y, Hiki N, Yoshikawa T, Kishi K, Ito Y, Ohi M (2015). Mediastinal lymph node metastasis and recurrence in adenocarcinoma of the esophagogastric junction. Surgery..

[31] Yamashita H, Katai H, Morita S, Saka M, Taniguchi H, Fukagawa T (2011). Optimal extent of lymph node dissection for Siewert type II esophagogastric junction carcinoma. Ann Surg..

[32] Peyre CG, Hagen JA, DeMeester SR, Altorki NK, Ancona E, Griffin SM (2008). The number of lymph nodes removed predicts survival in esophageal cancer: an international study on the impact of extent of surgical resection. Ann Surg..

[33] Pedrazzani C, de Manzoni G, Marrelli D, Giacopuzzi S, Corso G, Minicozzi AM (2007). Lymph node involvement in advanced gastroesophageal junction adenocarcinoma. J Thorac Cardiovasc Surg..

[34] Kurokawa Y, Takeuchi H, Doki Y, Mine S, Terashima M, Yasuda T (2019). Mapping of lymph node metastasis from esophagogastric junction tumors: a prospective nationwide multicenter study. Ann Surg.

[35] Harris A, Butterworth J, Boshier PR, MacKenzie H, Tokunaga M, Sunagawa H, et al. Development of a reliable surgical quality assurance system for 2-stage esophagectomy in randomized controlled trials. *Ann Surg.* 2020. 10.1097/SLA.0000000000003850 10.1097/SLA.0000000000003850 32224728

[36] Parry K, Haverkamp L, Bruijnen RC, Siersema PD, Ruurda JP, van Hillegersberg R (2015). Surgical treatment of adenocarcinomas of the gastro-esophageal junction. Ann Surg Oncol..

[37] Jezerskyte E, Saadeh LM, Hagens ERC, Sprangers MAG, Noteboom L, van Laarhoven HWM (2020). Long-term quality of life after total gastrectomy versus Ivor-Lewis esophagectomy. World J Surg..

[38] Kauppila JH, Ringborg C, Johar A, Lagergren J, Lagergren P (2018). Health-related quality of life after gastrectomy, esophagectomy, and combined esophagogastrectomy for gastroesophageal junction adenocarcinoma. Gastric Cancer..

[39] Fuchs H, Holscher AH, Leers J, Bludau M, Brinkmann S, Schroder W (2016). Long-term quality of life after surgery for adenocarcinoma of the esophagogastric junction: extended gastrectomy or transthoracic esophagectomy?. Gastric Cancer..

