# Supplementary material for: Esophagectomy or Total Gastrectomy for Siewert 2 Gastroesophageal Junction (GEJ) Adenocarcinoma? A Registry-Based Analysis
Source: Ann Surg Oncol. 2021 Jul 13;28(13):8485–94. doi: 10.1245/s10434-021-10346-x (PMC8591012; doi:10.1245/s10434-021-10346-x)
Supplement: Supplementary file 1 — Supplementary file1 (DOCX 34 kb) [file 10434_2021_10346_MOESM1_ESM.docx]

<TC>**TABLE S1** Summary of codes in the National Cancer Database used to derive the variables included in this study

| Tumor histology |  |
| --- | --- |
| Adenocarcinoma | 8140–8146 |
| Surgery codes |  |
| Esophagectomy | 30, 40, 50–55, 80 |

<TC>**TABLE S2** Summary table of variables used for propensity score-matching stratified by analysis

| Facility type | Race | Residence |
| --- | --- | --- |
| Facility location | CDCC score | Type of GEJ |
| Hospital distance | Surgical approach | AJCC clinical T classification |
| Year at diagnosis | Insurance status | AJCC clinical N classification |
| Age at diagnosis | Education level | Neoadjuvant therapy |
| Sex | Medical income |  |

CDCC, Charlson-Deyo comorbidity score ; GEJ, gastroesophageal junction; AJCC, American Joint Committee on Cancer

<TC>**TABLE S3** Logistic regression of factors associated with allocation to gastrectomy for patients with Siewert 2 GEJ adenocarcinoma

|  |  | Univariable OR (95 % CI) | Multivariable OR (95 % CI) |
| --- | --- | --- | --- |
| Facility type | Community | — | - |
|  | Integrated | 1.10 (0.89–1.38) (*p* = 0.383) | 1.16 (0.92–1.47) (*p* = 0.219) |
|  | Academic | 0.67 (0.58–0.77) (*p* < 0.001) | 0.77 (0.65–0.91) (*p* = 0.002) |
| Facility location | Northeast | — | — |
|  | Midwest | 0.82 (0.69–0.98) (*p* = 0.030) | 0.86 (0.71–1.04) (*p* = 0.114) |
|  | South | 1.46 (1.22–1.75) (*p* < 0.001) | 1.42 (1.16–1.73) (*p* = 0.001) |
|  | West | 1.01 (0.82–1.24) (*p* = 0.960) | 0.88 (0.70–1.10) (*p* = 0.251) |
| Hospital distance (miles) | <12.5 | — | — |
|  | 12.5–49.9 | 0.92 (0.79–1.07) (*p* = 0.273) | 1.05 (0.88–1.24) (*p* = 0.605) |
|  | ≥50 | 0.67 (0.57–0.80) (*p* < 0.001) | 0.91 (0.73–1.13) (*p* = 0.387) |
| Year of diagnosis | 2010–2011 | — | — |
|  | 2012–2013 | 0.66 (0.55–0.79) (*p* < 0.001) | 0.69 (0.58–0.84) (*p* < 0.001) |
|  | 2014–2015 | 0.82 (0.66–1.02) (*p* = 0.076) | 0.90 (0.72–1.13) (*p* = 0.368) |
|  | 2016–2017 | 0.68 (0.57–0.82) (*p* < 0.001) | 0.73 (0.60–0.88) (*p* = 0.001) |
| Age at diagnosis (years) | 18–35 | — | — |
|  | 36–50 | 0.42 (0.13–1.03) (*p* = 0.095) | 0.47 (0.14–1.23) (*p* = 0.170) |
|  | 51–65 | 0.32 (0.10–0.76) (*p* = 0.025) | 0.41 (0.12–1.03) (*p* = 0.091) |
|  | 66–80 | 0.42 (0.13–1.01) (*p* = 0.091) | 0.55 (0.16–1.41) (*p* = 0.265) |
|  | 80+ | 0.75 (0.22–2.03) (*p* = 0.610) | 0.78 (0.22–2.24) (*p* = 0.672) |
| Sex | Male | — | — |
|  | Female | 1.70 (1.40–2.08) (*p* < 0.001) | 1.57 (1.29–1.94) (*p* < 0.001) |
| Race | White | — | — |
|  | Other | 3.31 (2.30–4.96) (*p* < 0.001) | 3.11 (2.12–4.77) (*p* < 0.001) |
| CDCC score | 0 | — | — |
|  | 1–2 | 0.89 (0.77–1.02) (*p* = 0.103) | 0.86 (0.74–0.99) (*p* = 0.040) |
|  | 2+ | 1.65 (0.99–2.99) (*p* = 0.073) | 1.80 (1.05–3.37) (*p* = 0.046) |
| Insurance status | Medicare | — | — |
|  | Medicaid | 1.03 (0.77–1.42) (*p* = 0.848) | 1.11 (0.79–1.58) (*p* = 0.560) |
|  | Private | 0.85 (0.74–0.97) (*p* = 0.018) | 1.01 (0.83–1.21) (*p* = 0.956) |
|  | Not insured / Other | 2.07 (1.30–3.53) (*p* = 0.004) | 1.91 (1.17–3.32) (*p* = 0.014) |
| Education level (%) | >21 | — | — |
|  | 13–20.9 | 0.94 (0.77–1.14) (*p* = 0.517) | 1.05 (0.84–1.30) (*p* = 0.682) |
|  | 7–12.9 | 0.95 (0.79–1.14) (*p* = 0.566) | 1.11 (0.90–1.37) (*p* = 0.323) |
|  | <7 | 0.87 (0.72–1.06) (*p* = 0.180) | 0.93 (0.74–1.16) (*p* = 0.517) |
| Medical income ($) | ≤47,999 | — | — |
|  | 48,000–62,999 | 1.04 (0.88–1.24) (*p* = 0.631) | 1.12 (0.92–1.35) (*p* = 0.253) |
|  | 63,000 + | 1.09 (0.94–1.27) (*p* = 0.264) | 1.25 (1.02–1.53) (*p* = 0.030) |
| Residence | Metro | — | — |
|  | Urban | 0.78 (0.66–0.93) (*p* = 0.006) | 0.93 (0.76–1.15) (*p* = 0.516) |
|  | Rural | 0.69 (0.54–0.89) (*p* = 0.003) | 0.81 (0.62–1.06) (*p* = 0.117) |
| AJCC clinical T stage | cT1 | — | — |
|  | cT2 | 0.88 (0.73–1.07) (*p* = 0.211) | 1.00 (0.80–1.25) (*p* = 0.987) |
|  | cT3 | 1.16 (0.98–1.37) (*p* = 0.082) | 1.40 (1.12–1.74) (*p* = 0.003) |
|  | cT4 | 4.61 (2.21–11.81) (*p* < 0.001) | 4.49 (2.09–11.69) (*p* < 0.001) |
|  | cTx | 4.41 (3.30–5.97) (*p* < 0.001) | 3.73 (2.70–5.24) (*p* < 0.001) |
| AJCC clinical N stage | cN0 | — | — |
|  | cN1 | 0.90 (0.78–1.04) (*p* = 0.152) | 1.05 (0.89–1.24) (*p* = 0.546) |
|  | cN2 | 1.50 (1.14–2.00) (*p* = 0.005) | 1.68 (1.26–2.29) (*p* = 0.001) |
|  | cN3 | 1.64 (0.92–3.23) (*p* = 0.118) | 1.43 (0.79–2.87) (*p* = 0.272) |
|  | cNx | 3.80 (2.62–5.76) (*p* < 0.001) | 1.53 (1.00–2.41) (*p* = 0.059) |
| Neoadjuvant therapy | None | — | — |
|  | NCRT/NAC | 0.71 (0.62–0.82) (*p* < 0.001) | 0.71 (0.58–0.86) (*p* < 0.001) |
| Surgical approach | Open | — | — |
|  | Minimally Invasive | 0.66 (0.57–0.76) (*p* < 0.001) | 0.72 (0.62–0.84) (*p* < 0.001) |

GEJ, gastroesophageal junction; OR, odds ratio; CI, confidence interval; CDCC Charlson-Deyo comorbidity score; AJCC, American Joint Committee on Cancer; NCRT, neoadjuvant chemoradiotherapy; NAC, neoadjuvant chemotherapy

<TC>**TABLE S4** Sensitivity analysis of Cox regression of factors associated with survival for patients with Siewert 2 GEJ adenocarcinoma, accounting for pathologic tumor stage

|  |  | Univariable HR (95 % CI) | Multivariable HR (95 % CI) |
| --- | --- | --- | --- |
| Facility type | Academic | — | — |
|  | Community | 1.21 (1.11–1.33) (*p* < 0.001) | 1.14 (1.03–1.26) (*p* = 0.010) |
|  | Integrated | 1.20 (1.06–1.36) (*p* = 0.005) | 1.11 (0.97–1.26) (*p* = 0.125) |
| Facility location | Midwest | — | — |
|  | Northeast | 0.88 (0.78–0.98) (*p* = 0.020) | 0.92 (0.82–1.03) (*p* = 0.155) |
|  | South | 1.02 (0.92–1.14) (*p* = 0.710) | 1.03 (0.92–1.15) (*p* = 0.581) |
|  | West | 1.03 (0.91–1.16) (*p* = 0.669) | 1.05 (0.92–1.19) (*p* = 0.463) |
| Hospital distance (miles) | <12.5 | — | — |
|  | 12.5–49.9 | 1.11 (1.01–1.22) (*p* = 0.034) | 1.09 (0.98–1.21) (*p* = 0.099) |
|  | ≥50 | 1.22 (1.10–1.36) (*p* < 0.001) | 1.19 (1.04–1.36) (*p* = 0.009) |
| Year of diagnosis | 2010–2011 | — | — |
|  | 2012–2013 | 1.01 (0.91–1.12) (*p* = 0.825) | 1.02 (0.92–1.13) (*p* = 0.743) |
|  | 2014–2015 | 0.97 (0.85–1.11) (*p* = 0.667) | 0.97 (0.85–1.12) (*p* = 0.697) |
|  | 2016–2017 | 0.86 (0.76–0.97) (*p* = 0.016) | 0.86 (0.76–0.98) (*p* = 0.021) |
| Age at diagnosis (years) | 18–35 | — | — |
|  | 36–50 | 0.71 (0.41–1.22) (*p* = 0.211) | 0.85 (0.48–1.48) (*p* = 0.560) |
|  | 51–65 | 0.72 (0.42–1.21) (*p* = 0.213) | 0.89 (0.52–1.55) (*p* = 0.689) |
|  | 66–80 | 0.86 (0.50–1.45) (*p* = 0.563) | 1.04 (0.59–1.81) (*p* = 0.898) |
|  | 80+ | 1.17 (0.66–2.09) (*p* = 0.588) | 1.77 (0.97–3.23) (*p* = 0.064) |
| Sex | Female | — | — |
|  | Male | 1.12 (0.98–1.27) (*p* = 0.097) | 1.15 (1.01–1.31) (*p* = 0.040) |
| Race | Other | — | — |
|  | White | 0.96 (0.76–1.22) (*p* = 0.747) | 0.97 (0.76–1.24) (*p* = 0.815) |
| CDCC score | 0 | — | — |
|  | 1–2 | 1.14 (1.05–1.24) (*p* = 0.003) | 1.15 (1.05–1.26) (*p* = 0.002) |
|  | >2 | 1.09 (0.78–1.53) (*p* = 0.602) | 1.23 (0.87–1.72) (*p* = 0.237) |
| Insurance status | Medicare | — | — |
|  | Medicaid | 0.97 (0.80–1.17) (*p* = 0.732) | 1.01 (0.82–1.24) (*p* = 0.906) |
|  | Private | 0.80 (0.74–0.87) (*p* < 0.001) | 0.86 (0.77–0.96) (*p* = 0.009) |
|  | Not insured/other | 0.86 (0.70–1.07) (*p* = 0.170) | 0.92 (0.73–1.14) (*p* = 0.440) |
| Education level (%) | <7 | — | — |
|  | 7–12.9 | 1.00 (0.89–1.11) (*p* = 0.936) | 0.92 (0.82–1.04) (*p* = 0.167) |
|  | 13–20.9 | 0.99 (0.88–1.11) (*p* = 0.860) | 0.83 (0.72–0.96) (*p* = 0.010) |
|  | >21 | 0.80 (0.70–0.90) (*p* < 0.001) | 0.74 (0.64–0.85) (*p* < 0.001) |
| Medical income ($) | ≤47,999 | — | — |
|  | 48,000–62,999 | 0.89 (0.80–0.99) (*p* = 0.027) | 0.89 (0.79–0.99) (*p* = 0.035) |
|  | 63,000+ | 0.74 (0.67–0.82) (*p* < 0.001) | 0.76 (0.67–0.86) (*p* < 0.001) |
| Residence | Metro | — | — |
|  | Rural | 0.79 (0.67–0.94) (*p* = 0.006) | 0.78 (0.65–0.93) (*p* = 0.007) |
|  | Urban | 1.18 (1.06–1.31) (*p* = 0.002) | 1.01 (0.90–1.14) (*p* = 0.833) |
| AJCC pathological overall stage | 0 | — | — |
|  | 1 | 0.78 (0.68–0.90) (*p* < 0.001) | 0.92 (0.80–1.07) (*p* = 0.277) |
|  | 2 | 1.54 (1.32–1.80) (*p* < 0.001) | 1.59 (1.36–1.85) (*p* < 0.001) |
|  | 3 | 2.21 (1.96–2.51) (*p* < 0.001) | 2.37 (2.09–2.69) (*p* < 0.001) |
| Neoadjuvant therapy | None | — | — |
|  | NAC | 1.41 (1.23–1.61) (*p* < 0.001) | 1.23 (1.07–1.41) (*p* = 0.003) |
|  | NCRT | 1.49 (1.35–1.64) (*p* < 0.001) | 1.38 (1.24–1.54) (*p* < 0.001) |
| Surgical approach | Minimally invasive | — | — |
|  | Open | 1.12 (1.02–1.23) (*p* = 0.014) | 1.01 (0.92–1.12) (*p* = 0.762) |
| Type of surgery | Esophagectomy | — | — |
|  | Gastrectomy | 1.22 (1.10–1.36) (*p* < 0.001) | 1.12 (1.01–1.25) (*p* = 0.034) |

GEJ, gastroesophageal junction; HR, hazard ratio; CI, confidence interval; CDCC Charlson-Deyo comorbidity score; AJCC, American Joint Committee on Cancer; NAC, neoadjuvant chemotherapy; NCRT, neoadjuvant chemoradiotherapy
